# Generalized granuloma annulare after pneumococcal vaccination^☆^^☆☆^

**DOI:** 10.1016/j.abd.2020.05.009

**Published:** 2020-11-16

**Authors:** Miguel Fernando García-Gil, Marcial Álvarez-Salafranca, Alejandro Martínez García, Mariano Ara-Martín

**Affiliations:** a Department of Dermatology, Hospital Clínico Universitario Lozano Blesa, Zaragoza, Spain; b Department of Pathology, Hospital Clínico Universitario Lozano Blesa, Zaragoza, Spain

**Keywords:** Case reports, Granuloma annulare, Vaccination

## Abstract

Granuloma annulare may be caused by multiple triggers. Among these are vaccinations, which have been described as an infrequent cause of granuloma annulare. The authors report the first case of generalized granuloma annulare associated with pneumococcal vaccination in a 57-year-old woman, who presented cutaneous lesions 12 days after vaccination.

## Introduction

The occurrence of granuloma annulare (GA) triggered by vaccination is rare, and the mechanism by which it is triggered can be explained by the trauma of inoculation or by the immune mechanism involved in vaccination.^1^, ^2^, ^3^

## Case report

**Figure 1:**
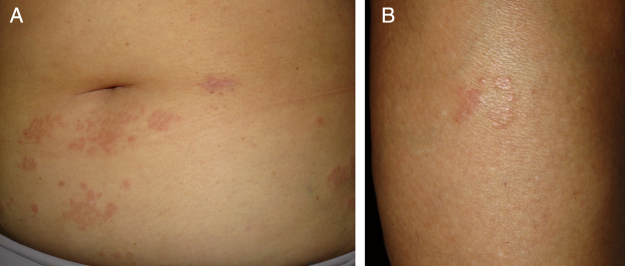
Annular erythematous-violaceous lesions on the abdomen and lower extremity.

**Figure 2:**
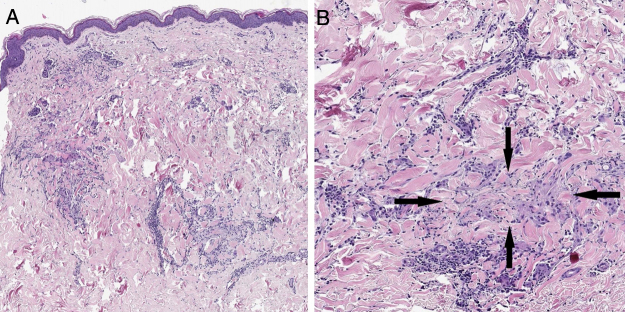
(A) A skin section showing the foci of chronic interstitial reticular dermis inflammation (Hematoxylin & eosin, ×20). (B) At a higher magnification, a clear necrobiosis of collagen fibers is observed (black arrows), along with an associated lymphohistiocytic infiltrate (Hematoxylin & eosin, ×40).

A 57-year-old woman with a medical history of hypothyroidism, hypercholesterolemia, and high blood pressure presented with a three-month history of asymptomatic skin lesions on the abdomen and lower extremities. Physical examination revealed multiple firm erythematous–violaceous papules clustered in an annular pattern and distributed along the abdomen and lower extremities (Fig. 1). A 13-valent pneumococcal conjugate vaccine (PCV-13) was identified as a possible trigger, since it had been administered 12 days prior to the onset of the skin lesions. A skin biopsy of the abdominal lesion was performed. Histopathological examination revealed foci of chronic interstitial inflammation and collagen necrobiosis with an associated lymphohistiocytic infiltrate (Fig. 2).

The patient was diagnosed with generalized GA following pneumococcal vaccination based on the clinical appearance of the lesions, histopathological findings, and temporal relationship between the vaccinations and skin lesions. Treatment using mometasone furoate cream was initiated; however, no improvement was apparent after one month. The lesions eventually resolved spontaneously within two months of their initial appearance.

## Discussion

Only 13 cases of GA following vaccination have been reported in the literature. The bacillus Calmette-Guérin (BCG) vaccine has been most frequently reported to be causative vaccine (eight cases), followed by the hepatitis B vaccine (HBV; two cases) and the influenza, tetanus and diphtheria–tetanus toxoid vaccines (one case each).^1^, ^2^, ^3^, ^4^, ^5^, ^6^, ^7^, ^8^, ^9^, ^10^ An association between the pneumococcal vaccine and GA has not been previously described (Table 1).

**Table 1:** Cases of granuloma annulare triggered by vaccination.

Authors	Year	Vaccine	Dose	Sex	Age	Localized/generalized	Latency time	Treatment	Resolution	Revaccination recurrence
Houcke-Bruge C et al.	2001	BCG	1 dose	Male	3 years	Generalized	1 month	No	Complete, 3 months	
		BCG	1 dose	Female	2 years	Location remote from vaccination site	2 months	No	Complete, 3 months	
Kakurai M et al.	2001	BCG	1 dose	Male	12 years	Generalized	5 days	No	Complete, 7 months	
Nagase K et al.	2011	BCG	1 dose	Male	5 months	Generalized	1 month	Moisturizer, 3 months	Complete, 3 months	
Lee SW et al.	2011	BCG	1 dose	Female	3 months	Generalized	1 month	Topical corticosteroids, 1 month	Complete, 1 month	
Nomiyama T et al.	2013	BCG	1 dose	Female	6 months	Generalized	1 month	Topical corticosteroids 1 month	Complete, 1 month	
Yoon NY et al.	2014	BCG	1 dose	Male	3 months	Generalized	7 weeks	Ceftriaxone for 8 days and prophylactic hydroxyzine for 3 weeks	Complete, 3 weeks	
Yang SY et al.	2018	BCG	1 dose	Male	3 months	Generalized	2 months	No	Complete, 18 months	
Wolf F et al.	1998	Hepatitis B virus	4 doses (0 months, 1 month, 2 months, 12 months)	Female	51 years	Generalized	1 month after the last vaccine	Dapsone 50 mg/day 4 months	Complete, 4 months (subsequent recurrence)	Revaccination at 5 years (latency time of 3 weeks)
Criado PR et al.	2004	Hepatitis B virus	2 doses (0 months, 1 month)	Female	58 years	Generalized	2 months after the last vaccine	Topical corticosteroids	Partial improvement, 3 months	
Baykal C et al.	2002	Tetanus	1 dose	Female	6 years	Generalized	2 months	Triamcinolone acetonide 10 mg/mL every 4 weeks, intralesional (3 sessions)	Complete, 3 months (subsequent recurrence)	Revaccination (latency time of 3 days)
Baskan EB et al.	2005	Diphtheria and tetanus toxoid	1 dose	Female	8 years	Generalized	1 week	Topical corticosteroids	No improvement	
Suzuki T et al.	2014	Influenza	1 dose	Female	76 years	Generalized	1 month	No	No follow-up	

PCV-13, 13-valent pneumococcal conjugate vaccine; BCG vaccine, Bacillus Calmette-Guérin vaccine.

The occurrence of GA triggered by vaccination has been reported to be higher in females (61.54%, eight cases) than in males (38.46%, five cases). The generalized form (76.92%, ten cases) is the most frequently described clinical form of GA triggered by vaccination, with the localized form accounting only for 23.08% of the cases (3 cases). The ages of the affected patients ranged from 3 months to 76 years (average, 16.74 years; median, 3 years). The latency between vaccination and the appearance of GA has been reported to range from 5 days to 2 months (average, 1.22 months; median, 1 month).

GA completely healed in most reported cases (10 cases).^1^, ^2^, ^4^, ^5^, ^6^, ^7^ However, partial improvement was observed in one case, while no improvement was observed in another case.^3^, ^8^ Overall, the mean resolution time was 4.37 months in the completely healed cases (median, 3 months). GA recurred following re-vaccination with the HBV and tetanus vaccines in two cases, with a latency of 3 weeks in both the cases.^7^, ^10^ The latency to recurrence was thus shorter in the cases with re-vaccinations.

The mechanism by which GA is triggered is unknown. Immunological activation after vaccination would explain the presence of activated T-cells in the lymphocytic infiltrate of GA, suggesting the existence of a mediated immune response.

GA triggered by vaccination could have an immunological pathogenesis, as generalized GA has been observed in most cases. The traumatic inoculation hypothesis is less convincing, because GA exclusively located at the vaccination site has been observed in few cases.^6^, ^9^

The short latency between vaccination and the appearance of GA, which has been reported to be around 1 month in most cases, supports the causal relationship between vaccination and GA. Recurrence following re-vaccinations and the concomitant shorter latency further support this argument. After the cessation of immunological stimulation, GA healed completely in most cases, including in the present case.^1^, ^2^, ^4^, ^5^, ^6^, ^7^, ^10^ Notably, most cases occurred in young patients, probably because the frequency of vaccination is higher at a younger age. However, cases have also been observed in older patients.

In conclusion, the majority of reported cases of GA are of the generalized form, which indicates a possible immunological pathogenesis. Complete resolution was observed within a few months after vaccination in most cases, and recurrence occurred on re-vaccination.

## Financial support

None declared.

## Authors' contributions

Miguel Fernando García-Gil: Statistical analysis; approval of the final version of the manuscript; design and planning of the study; drafting and editing of the manuscript; collection, analysis, and interpretation of data; effective participation in research orientation; intellectual participation in the propaedeutic and/or therapeutic conduct of the studied cases; critical review of the literature; critical review of the manuscript.

Marcial Álvarez-Salafranca: Statistical analysis; approval of the final version of the manuscript; design and planning of the study; drafting and editing of the manuscript; collection, analysis, and interpretation of data; effective participation in research orientation; intellectual participation in the propaedeutic and/or therapeutic conduct of the studied cases; critical review of the literature; critical review of the manuscript.

Alejandro Martínez García: Statistical analysis; approval of the final version of the manuscript; design and planning of the study; drafting and editing of the manuscript; collection, analysis, and interpretation of data; effective participation in research orientation; intellectual participation in the propaedeutic and/or therapeutic conduct of the studied cases; critical review of the literature; critical review of the manuscript.

Mariano Ara-Martín: Statistical analysis; approval of the final version of the manuscript; design and planning of the study; drafting and editing of the manuscript; collection, analysis, and interpretation of data; effective participation in research orientation; intellectual participation in the propaedeutic and/or therapeutic conduct of the studied cases; critical review of the literature; critical review of the manuscript.

## Conflicts of interest

None declared.
